# Recent clinical studies of the immunomodulatory drug (IMiD®) lenalidomide

**DOI:** 10.1038/sj.bjc.6602774

**Published:** 2005-09-06

**Authors:** J B Bartlett, A Tozer, D Stirling, J B Zeldis

**Affiliations:** 1 Celgene Corporation, 86 Morris Avenue, Summit, NJ 07901, USA

**Keywords:** lenalidomide, thalidomide, multiple myeloma, myelodysplastic syndromes, immunomodulatory, angiogenesis

## Abstract

Thalidomide is effective in the treatment of multiple myeloma. The immunomodulatory drug and thalidomide analogue lenalidomide is currently in late stage clinical development for MDS and multiple myeloma. This minireview highlights the course of initial and ongoing lenalidomide clinical development in oncology with reference to earlier thalidomide studies.

## PRECLINICAL DEVELOPMENT OF IMiDS®

The IMiDs® are immunomodulatory thalidomide analogues designed to optimise certain properties including anticancer properties, but they lack or minimise much of the toxicity associated with thalidomide (Bartlett *et al*, 2004a). Extensive preclinical testing of this class of compound, involving pharmacology, pharmacokinetics and toxicity, has led to the identification of lenalidomide (CC-5013; REVLIMID®) for clinical development in the oncology setting.

While the IMiDs share many of thalidomide's biological properties, the relative potency and range of these effects vary quite dramatically from each other and from thalidomide. Thus, each molecule cannot be assumed to have the same overall biological effects or therapeutic properties as thalidomide or other IMiDs. Preclinical data continue to support the clinical application of lenalidomide in cancer. For example, in contrast to thalidomide's teratogenicity, lenalidomide is nonteratogenic in the New Zealand rabbit preclinical model, which is the most sensitive animal model for thalidomide-associated teratogenicity.

The IMiDs have many different effects on biological systems and the combination of these properties probably accounts for their antineoplastic activity. IMiDs are antiangiogenic (Dredge *et al*, 2002a; Lentzsch *et al*, 2002) and T-cell costimulatory (Haslett *et al*, 1998). IMiDs also activate the innate component of the immune system: enhanced natural killer cell cytotoxicity leading directly to multiple myeloma cell lysis (Davies *et al*, 2001). The ability of IMiDs to activate innate immune responses may be crucial to the generation of effective adaptive antitumour responses *in vivo*, although this area remains relatively unexplored, as has a potential role for IMiDs in the enhancement of protective antitumour immunity (Dredge *et al*, 2002b).

Certain IMiDs appear to possess direct *in vitro* antimyeloma activity (Hideshima *et al*, 2000); primary human multiple myeloma (MM) cells derived from the bone marrow of patients resistant to chemotherapy are susceptible to IMiD-induced growth arrest. IMiDs may also potentiate the effects of TNF-related apoptosis-inducing ligand (TRAIL), dexamethasone and proteasome inhibitors that are currently used as antimyeloma therapies. These IMiDs can also interfere with myeloma cell–bone marrow stromal cell interactions by affecting adhesion molecule expression, which might be crucial for MM cell growth and survival, and prevent the upregulation of IL-6 and vascular endothelial growth factor (VEGF), which is involved in angiogenesis (Gupta *et al*, 2001).

## CLINICAL DEVELOPMENT OF LENALIDOMIDE

Thalidomide is currently indicated in the US only for the cutaneous manifestations of erythema nodosum leprosum (ENL), a reactional state of leprosy. A regulatory application is under review by the US Food and Drug Administration for use in MM. In Australia, New Zealand, Turkey and Israel, thalidomide is approved for the treatment of MM after the failure of standard therapies, as well as for ENL. Teratogenicity is the most serious potential side effect; therefore, thalidomide is available only under a restricted distribution system. In order to understand how IMiDs with their broad preclinical activities might affect human disease, Celgene embarked on a strategy to evaluate thalidomide's therapeutic activity in small groups of patients with a wide variety of diseases. Larger trials in more targeted disease states followed, based on the early signals obtained during these small clinical trials. Discussed below will be a brief review of what has been observed with thalidomide and lessons learned for further development of lenalidomide, the lead IMiD chosen for use in the oncology setting.

### Multiple myeloma

Multiple myeloma is an incurable B-cell malignancy characterised by the clonal proliferation of malignant cells, usually in the bone marrow, that leads to the production of a monoclonal immunoglobulin. Multiple myeloma afflicts 14 000–15 000 new patients annually in the US. The current median survival rate for symptomatic patients is 3–5 years. High-dose chemotherapy (Melphalan) combined with transplantation of haematopoietic stem cells increases the rate of complete remission and extends event-free and overall survival. However, relapse rates are very high and few salvage therapies are available. Antiangiogenic therapy is a viable option for the treatment of patients with MM since it is associated with prominent bone marrow vascularity, the degree of which correlates with prognosis. In addition, plasma levels of various proangiogenic molecules are elevated in active MM. It is also evident that circulating endothelial progenitor cells (EPC) contribute to angiogenesis in MM and that the elevation of EPC levels correlates with disease activity. Interestingly, inhibition of EPC is associated with patient response to thalidomide (Zhang *et al*, 2005). Also, since adhesion to bone marrow stroma is associated with chemotherapy resistance, thalidomide and lenalidomide therapy might theoretically make previously chemotherapy-resistant tumours sensitive by affecting stromal–myeloma cell interactions. The clinical strategy for the use of thalidomide in MM was to determine its activity as monotherapy, followed by trials using it with other drug combinations including drugs that were not previously effective in treating patients.

Monotherapy thalidomide trials in advanced relapsed/refractory (Kumar *et al*, 2003; Richardson *et al*, 2004) as well as previously untreated asymptomatic MM (Rajkumar *et al*, 2003; Weber *et al*, 2003) (also known as ‘smouldering’ or indolent myeloma) have consistently shown partial response rates (SWOG criteria) of approximately 30%, with an acceptable safety profile. Thalidomide combined with dexamethasone in relapsed, refractory MM induces a partial response in 56% of patients (Palumbo *et al*, 2004a). In newly diagnosed patients, thalidomide and dexamethasone therapy resulted in 64% partial responses (Rajkumar *et al*, 2002). A planned interim analysis of a phase III Eastern Cooperative Oncology Group study in newly diagnosed MM patients demonstrated a significantly higher response rate in patients randomised to thalidomide with dexamethasone than in those on dexamethasone alone (80 *vs* 53%, respectively, *P*=0.0023, *n*=109). Grade 3–4 toxicities were higher in the combination therapy arm, particularly deep vein thrombosis (Rajkumar *et al*, 2004a). The results of this ECOG trial have been submitted to the FDA to support the supplemental New Drug Application (sNDA) for the use of thalidomide in the treatment of MM.

Although uncommon with single agent thalidomide, there may be potential for thromboembolic events when thalidomide is combined with dexamethasone and other agents, which has led to preliminary evaluation of various regimens as prophylaxis in high-risk patients. Full therapeutic dose anticoagulation (either coumadin or low molecular weight heparin) has been used in patients who are at higher risk for DVT, with some evidence of prophylactic effect (Zangari *et al*, 2001) and aspirin may have a role in reducing incidence associated with thalidomide and DVd (Baz *et al*, 2004). While being anticoagulated as a treatment for a DVT, patients can be continued on thalidomide treatment (Zangari *et al*, 2001).

Encouraging results have also been observed with thalidomide when combined with a corticosteroid and a chemotherapeutic agent. A variety of such combinations of oral medications in newly diagnosed patients have produced greater than 80% partial responses and up to 26% complete responses (Dimopoulos *et al*, 2004; Palumbo *et al*, 2004b). Increased toxicities, including venous thromboses, have been seen with the melphalan/prednisone/dexamethasone (MPT) regimen (neither the pulsed melphalan/dexamethasone/thalidomide (MDT) nor the cyclophosphamide/thalidomide/dexamethasone (CTD) regimen reported increased DVT incidence), suggesting that the sequential administration of chemotherapy and thalidomide may result in higher responses, without an increase in DVTs (Dimopoulos *et al*, 2004). Thalidomide's addition to other commonly used combination therapies, such as VAD or Doxil/Vincristine/dexamethasone (DVd), results in almost a doubling of the response and complete response rate. Increased venous thromboses and infections may be controlled or prevented with low molecular heparin or low-dose aspirin and antibiotics, respectively.

Thalidomide has been observed to have activity in other haematological diseases and cancers, including myelodysplastic syndromes, MDS (Raza *et al*, 2001), chronic lymphocytic leukaemia (Furman *et al*, 2005), myelofibrosis (Mesa *et al*, 2004), Waldenstrom's macroglobulinaemia (Dimopoulos *et al*, 2003), prostate cancer (Dahut *et al*, 2004) and brain cancer (Fine *et al*, 2003).

On the basis of the thalidomide experience, lenalidomide was evaluated as an oral MM treatment in two Phase III clinical studies in patients with relapsed, refractory MM. Thalidomide-like side effects, such as somnolence, constipation or neuropathy were of low incidence despite anti-MM activity. At least 71% of 24 relapsed and refractory patients (median of three prior treatment regimens including autologous stem cell transplant and thalidomide in 15 and 16 patients, respectively) evaluated in a dose-finding trial experienced ⩾25% paraprotein reduction. The median time to best response was 2 months and the median treatment duration 6 months (Richardson *et al*, 2002). The most common grade 3 and 4 adverse events were neutropenia (grade 3 in 60%; grade 4 in 16%) and thrombocytopenia (grade 3 in 20%). No thromboembolic events were reported in this first monotherapy trial of lenalidomide in relapsed, refractory myeloma.

Other studies evaluated different dosing regimens in order to minimise myelosuppression. A phase II study comparing 25 mg lenalidomide daily for 20 days *vs* 50 mg daily for 10 days (both regimens repeated every 28 days) in relapsed, refractory MM patients reported that the 25 mg dosing schedule produced a greater response rate. A total of 40% of patients achieved ⩾50% paraprotein reduction with this dosing *vs* 15% using the higher dose/shorter regimen. The estimated 12-month event-free survival was 30%, and the estimated overall survival was 61% (Zangari *et al*, 2003).

A larger study evaluated 83 patients on one of two syncopated dosing schedules, and found that 30 mg daily (21 days out of every 28 days) caused less myelosuppression than 15 mg b.i.d. (21 days out of every 28), although response rates were similar. A total of 24% of patients achieved ⩾50% paraprotein reduction, including five patients with complete remission (6%). An additional 33% of 30 patients who progressed on lenalidomide alone achieved at least a partial response (⩾50% paraprotein reduction) when dexamethasone was added (Richardson *et al*, 2003).

Lenalidomide has also been evaluated with Doxil, Vincristine and dexamethasone (DVd) in relapsed, refractory MM (Baz *et al*, 2004) and in combination with dexamethasone in newly diagnosed MM (Rajkumar *et al*, 2004b). A phase I/II trial of lenalidomide in refractory MM in combination with DVd (DVd-R) reported a 33% (seven out of 21) complete and near complete response and a 66% SWOG response. With prophylactic low-dose aspirin, one pulmonary embolus occurred (in a refractory patient with renal failure, performance status 3). Amoxicillin and acyclovir were also included in the protocol. Grade 3 neutropenia occurred in two patients, but there was no neutropenic sepsis. The other grade 3 adverse event reported was neuropathy (one patient) (Hussein *et al*, 2004).

The first report of lenalidomide in newly diagnosed MM patients was in combination with pulsed dexamethasone. The first analysis of this phase II trial occurred when the trial had been running for less than 6 months. Despite this short observation period, an 83% (25 out of 30) objective response rate was observed. Patients received 81 mg day^−1^ of aspirin as DVT prophylaxis. Although various grade 3 nonhaematological toxicities were observed, there were no toxicities ⩾grade 4 and no deep vein thromboses (Rajkumar *et al*, 2004b).

Two phase III randomised, double-blind, multicentre trials (US, Canada, Australia and Europe) of lenalidomide plus high-dose dexamethasone *vs* high-dose dexamethasone alone in previously treated patients with MM were conducted in more than 700 relapsed/refractory patients. In March 2005, a protocol-specified interim analysis by an Independent Data Monitoring Committee determined that for both trials there was a statistically significant improvement in time to disease progression in those patients randomised to receive lenalidomide/dexamethasone *vs* dexamethasone alone. The prespecified *P*-value for stopping the trials (*P*<0.0015) was exceeded and the safety profile was favourable. Reported in a Scientific Symposium at the 2005 American Society of Clinical Oncology (ASCO), the primary end point of time –to disease progression in the lenalidomide plus dexamethasone arm was 60.1 weeks (53.4 weeks – International) compared with a median time –to disease progression of 20.7 weeks (20.6 – International) for the dexamethasone alone arm (*P*⩽0.00001). Overall response rate with lenalidomide/dex was 61.2% (57.9% – International) *vs* 22.8% (21.7% – International) with dex alone (*P*⩽0.001). The complete response rate was 26.5 and 13.6% (lenalidomide/dex) *vs* 4.1 and 4.0% (dex alone) based on the investigators’ assessment.

Increased side effects were noted in the combination arm, compared with the dex/placebo arm, particularly grade 3/4 neutropenia and thrombocytopenia. Thromboembolic events were noted in a greater percentage of US patients than International patients (13.5 and 4.5%, respectively) (*vs* 3.5/3.4% in placebo/dex arm), suggesting that further investigation is required to explain this finding.

Treatment assignments will be unblinded, affording patients in the control arm the opportunity to add lenalidomide to their treatment regimen. These trials will form the basis for regulatory filings.

Lenalidomide is also currently used in combination with other therapies that have antimyeloma activity. A number of cooperative groups in US and Europe are evaluating lenalidomide's activity in a variety of MM disease states (See Table 1).

### MDS and other haematological malignancies

The myelodysplastic syndromes (MDS) are a spectrum of malignant disorders of blood cell production, which affects over 250 000 people worldwide, with the potential to develop into acute leukaemia. Lenalidomide appears to be very active in restoring red blood cell production in anaemic MDS patients, enabling them to become independent of blood transfusions. Even more remarkable is the effect of lenalidomide on patients with the 5q-syndrome (a type of MDS) in which treatment can render undetectable the 5q-chromosomal deletion that defines the condition. The apparent efficacy of lenalidomide in this patient population led to the submission of a New Drug Application (NDA) in April 2005 seeking approval to market this drug as a treatment for transfusion-dependent MDS patients with a 5q deletion chromosomal abnormality.

In an open-label trial, 43 patients with primary MDS (French–American–British (FAB) criteria), who were transfusion dependent (74%) or had symptomatic anaemia (Hg <10g dl^−1^), received lenalidomide for 16 weeks (List *et al*, 2005a). Three oral dosing schedules were evaluated: 25, 10 and 10 mg day^−1^ × 21 days of a 28-day cycle. Patients had derived no response to recombinant erythropoietin, or had a low probability of responding (endogenous serum level >500 mU ml^−1^). FAB classes were refractory anaemia (RA-47%), RA with ringed sideroblasts (RARS-30%), RA with excess blasts (RAEB-19%), RAEB in transformation (RAEB-t-2%) and chronic myelomonocytic leukaemia (CMML-2%). Clonal karyotypic abnormalities (⩾2 abnormal cells in metaphase) were present in 46% of patients, including interstitial deletion of chromosome 5q31.1 alone in 11 patients.

Response rate (modified International Working Group (IWG) criteria) was 56%, with 20 out of 32 (63%) transfusion-dependent patients achieving transfusion independence, and one out of 11 patients, who did not require transfusions, increasing haemoglobin >2 g dl^−1^. The median time to response varied with dose level (9 weeks at 25 mg day^−1^ to 11.5 weeks at 10 mg syncopated dosing). After a median of 81 weeks, the median duration of transfusion independence had not been reached and the median haemoglobin was 13.2 g dl^−1^.

An interesting finding was that the cytogenetic pattern was significantly correlated with haematological response: 83% of patients with 5q31.1 deletion responded *vs* 57% with a normal karyotype and 15% with other cytogenetic abnormalities. FAB and IPSS categories did not correlate with response. The median time to response was more rapid in patients with 5q31.1 deletion than in patients with a normal or other karyotype (8 weeks *vs* 11.2, *P*=0.029). Eleven out of 20 patients with clonal cytogenetic abnormalities had cytogenetic responses, including 10 complete cytogenetic remissions. Nine of the 10 complete cytogenetic remissions occurred in patients with del(5)(q31.1). The median time to cytogenetic remission was 8 weeks.

Neutropenia (65%, all ⩾grade 3) and thrombocytopenia (74% overall; 54% ⩾grade 3) were the most common adverse events. Severe myelosuppression was dose dependent and led to treatment interruption or dose reduction in 58% of patients with the median time to treatment resumption being 22 days. Other adverse events were mild and infrequent. This initial indication of lenalidomide activity in MDS led to phase II and III studies of lenalidomide in MDS with and without 5q deletion.

Data reported in a plenary session at ASCO 2005 support the previous findings. Of 148 transfusion-dependent MDS patients with del5q31, participating in a multicentre phase II trial of lenalidomide, 66% achieved transfusion independence (ITT analysis), achieving a median haemoglobin increase of 5.3 g dl^−1^. With a median duration of 58 weeks follow-up, 73% of responders remained transfusion independent, and the median duration of transfusion independence had not been reached. A greater proportion of patients with an isolated, *vs* other del5q became transfusion independent (69 *vs* 49%, *P*=0.003). Cytogentic response was noted in 70% of patients; 63% of these achieved complete cytogenetic remission. The most common adverse events were ⩾grade 3 neutropenia or thrombocytopenia, which resolved after dose reduction or after temporarily withholding lenalidomide. In total, 15 deaths occurred, two of which were suspected to be drug related (List *et al*, 2005b). The data from this trial form the basis for the NDA.

Lenalidomide is currently in phase II studies in myelofibrosis with myeloid metaplasia. Clinically relevant responses have been reported in four out of 15 (27%) patients. Improvement in anaemia (two patients), splenomegaly (two patients) and/or constitutional symptoms (three patients) occurred. One patient became transfusion independent in addition to experiencing marked improvement in constitutional symptoms. One patient's haemoglobin increased to 13.4 g dl^−1^ (from 8.3 g dl^−1^), leucocytes decreased from >66 × 10^9^ to within normal range and circulating blasts disappeared (pretreatment level 9%) after lenalidomide therapy. Grade 3 and 4 adverse events included neutropenia (five patients), thrombocytopenia (three patients), rash and fatigue (two patients each). Three patients experienced extreme thrombocytosis (Tefferi *et al*, 2004) (steep increase in platelet count has also been reported in a small number of patients taking thalidomide for myelofibrosis). One patient developed disseminated extramedullary haematopoiesis after taking lenalidomide for 1 month.

Encouraging results have also been reported from a small trial of lenalidomide in chronic lymphocytic leukaemia, with six out of seven evaluable patients responding at day 30 (one CR, five PR, one withdrew consent) (Miller *et al*, 2005). Lenalidomide is also currently being evaluated in other haematological conditions including amyloidosis and Waldenstrom's macroglobulinaemia.

Lenalidomide has been granted both Orphan Drug and Fast Track status by the FDA and Orphan Drug status for both MM and MDS by the European Commission on MM and the European Commission on MDS.

### IMiDs in solid tumours

The first published study of lenalidomide in patients with solid tumours suggests that lenalidomide has limited clinical activity in patients with advanced and heavily pretreated metastatic malignant melanoma and other solid cancers (Bartlett *et al*, 2004b). The primary objective of this phase I study was to assess the safety and tolerability of lenalidomide and in this regard there were no serious adverse effects attributed to treatment. Also, analyses of serum cytokines and peripheral blood cell-surface markers showed conclusive evidence for immune activation. However, unblinded interim results of two phase III lenalidomide monotherapy studies in malignant melanoma patients determined that a statistically significant treatment effect would not be achieved, although the safety profile was acceptable. On the recommendation of the Data Monitoring Committee, the programme was halted.

Lenalidomide is currently being evaluated in non-Hodgkin's lymphoma, NSCLC and cancers of the pancreas, prostate, brain, kidneys and ovaries.

## CONCLUSIONS

Thalidomide is now recognised as an agent with meaningful activity in haematology and oncology. This activity provided the rationale for the development of thalidomide analogues with the goal of enhancing antineoplastic activity while reducing or eliminating thalidomide-associated side effects. The IMiDs® have antiangiogenic, direct antitumour, anti-inflammatory and immune stimulatory properties. A particular challenge is to characterise how these properties interact within the physiological milieu of disease states.

As this review has documented, lenalidomide has entered the clinic for the treatment of various cancers. Pharmacological, preclinical and clinical data clearly demonstrate qualitative and quantitative differences between thalidomide and lenalidomide. The reported remarkable safety and efficacy of lenalidomide in the treatment of MDS and MM support regulatory filings in these indications. In addition, other neoplastic conditions will be assessed for sensitivity to lenalidomide. As with most agents in haematology/oncology, the authors believe that appropriate combination therapy involving lenalidomide will lead to highly effective therapy for treating a variety of malignant conditions. We look forward to understanding how various combination therapies can improve the management of afflicted patients.

## Figures and Tables

**Table 1:** Completed and ongoing clinical studies of Lenalidomide

**Drug**	**Indication**	**Country**	**Phase**	**Status**	**Comments and references**
*Multiple myeloma, monotherapy*					
Lenalidomide	Multiple myeloma, relapsed (*n*=27)	USA	I	Completed	First published report in MM (Richardson *et al*, 2002). A dose-escalating study with 24 evaluable patients. Best responses in terms of reduction in serum M-protein in evaluated patients were >50% in seven out of 24 (30%), >25–50% in 10 out of 24 (42%), <25% in two out of 24 (8%) patients. The maximum tolerated dose was 25 mg day^−1^. Grade 3 myelosuppression was apparent with patients treated with 50 mg day^−1^. No somnolence or neuropathy was observed.
Lenalidomide±Dex	Multiple myeloma, relapsed/refractory (*n*=102)	Multicentre, USA	II	Completed	Preliminary data from American Society of Hematology (ASH 2003) (Richardson *et al*, 2003) comparing two dose levels (15 mg b.i.d. *vs* 30 mg qd × 3 weeks with 1 week rest) of lenalidomide±dexamethasone. Overall response rate was 38%, including 6%CR and 18%PR. In all, 33% of patients who received dex for progressive disease achieved ⩾PR. Significant toxicities were thrombocytopenia (18%) and neutropenia (28%). Lower frequency of myelosuppression with the 30 mg qd cohort.
Lenalidomide	Multiple myeloma, relapsed/refractory (*n*=224)	Multicentre, USA	II	Ongoing	Closed to enrolment. Preliminary data from International Myeloma Workshop (IMW) 2005 (Haematologica/The Hematology Journal; 90 (Suppl 1): 154, Abstract # PO.737) demonstrated 25% paraprotein reduction in 28% of patients with advanced MM and poor prognosis. Most common grade 3 or 4 adverse events were neutropenia and thrombocytopenia.
Lenalidomide	Multiple myeloma, relapsed/refractory (*n*=100)	USA	II	Ongoing	Closed to enrolment. Very early data on the first 38 patients (IMW 2003; Hematology Journal; 4(Suppl 1): S5–S7) reported that lenalidomide at 50 mg qd × 10 days repeated q 28 days appeared inferior in terms of response (compared to 25 mg qd × 20) prompting dose modification to 50 mg qod^−1^ × 10. No significant sedation or neurotoxicity was observed. Myelosuppression was dose limiting.
					
*Multiple myeloma, combination therapy*					
Lenalidomide+bortezomib	Multiple myeloma, relapsed/refractory (*n*=58)	USA	I	Ongoing	Preliminary data from European Haematology Association (EHA) 2005 (Haematologica/The Hematology Journal 2005; 90 (Suppl 1): 26–27, Abstract # PL5.04) on the first nine patients treated: MTD was not reached in the first three cohorts. With a median of six cycles completed, patients have tolerated bortezomib 1.0–1.3 mg m^−2^ and lenalidomide 5–10 mg day^−1^ without DLT. All nine patients achieved minor response or stable disease. Grade 4 neutropenia (two patients, <5 days duration) and grade 3 thrombocytpopenia was reported in four patients.
Lenalidomide+DVd (DVd-R) Doxil, Vincristine, dexamethasone-Revlimid	Multiple myeloma, advanced relapsed/refractory (*n*=55)	USA	I/II	Ongoing	Preliminary results from 2004 ASH meeting (Hussein *et al*, 2004) report that DVd-R is an extremely effective regimen in refractory stage III MM. In 21 evaluable patients, the response rate (SWOG) was >66%, including CR+near CR rate of 33%, with minimal toxicity. Maximum tolerated dose defined as 10 mg qd × 21, q 28 days. The DLTs included sepsis/shock (at 15 mg), non-neutropenic sepsis (two patients), PE (one patient) and grade 3 neutropenia (two patients) and neuropathy (one patient).
Lenalidomide plus dexamethasone	Multiple myeloma, newly diagnosed (*n*=34)	USA	II	Completed	Preliminary results presented at 2004 American Society of Hematology (Rajkumar *et al*, 2004a, 2004b) suggest that lenalidomide /dexamethasone offers clinical benefit and is well tolerated in newly diagnosed MM. Objective responses were reported in 25 out of 30 patients (83%). Daily aspirin was given, no DVTs were reported. No grade⩾4 toxicities were reported. A total of 33% experienced various grade 3 toxicities.
Lenalidomide and dexamethasone *vs* dexamethasone alone	Multiple myeloma, refractory (*n*=354)	Multicentre, USA/Canada	III	Ongoing	Closed to enrolment. Results presented at the 2005 EHA (Haematologica/The Hematology Journal 2005; 90 (Suppl 2): 160, Abstract # 0402). Planned interim analysis by Independent Data Monitoring Committee showed statistically significant increase in TTP in the lenalidomide/dex arm (not reached at 60 weeks) *vs* 19.9 weeks in dex only arm. Overall response rate: 51.3% (len/dex) *vs* 22.9% (dex); CR assessed by investigator: 19.5% (len/dex) *vs* 3.8% (dex).Well tolerated.
Lenalidomide and dexamethasone *vs* dexamethasone alone	Multiple myeloma, refractory (*n*=351)	Multicentre, International	III	Ongoing	Closed to enrolment. Planned interim analysis by Independent Data Monitoring Committee showed statistically significant increase in the TTP in lenalidomide/dex arm (not reached at 47 weeks) *vs* 20.4 weeks in dex only arm (Dimopoulos *et al*, 2005). Overall response rate: 47.6% (len/dex) *vs* 18.4% (dex); CR assessed by investigator: 9.1% (len/dex) *vs* 1.2% (dex). Well tolerated.
Lenalidomide and dexamethasone *vs* dexamethasone alone	Multiple myeloma, newly diagnosed (*n*= 500)	USA, National Cancer Institute (NCI) and Southwest Oncology group (SWOG), USA	III	Ongoing	No data reported as yet
Lenalidomide+dexamethasone *vs* lenalidomide+low-dose dexamethasone	Multiple myeloma (*n*=412)	USA, Eastern Cooperative Oncology Group (ECOG)	III	Ongoing	No data reported as yet
Lenalidomide+bortezomib *vs* bortezomib	Multiple myeloma, relapsing/progressing on total therapy III (*n*=315)	USA	III	Ongoing	No data reported as yet
					
*Myelodysplastic syndromes*					
Lenalidomide	Myelodysplastic syndromes with 5q− cytogenetic abnormality (*n*=151)	Multicentre, USA	II	Ongoing	Closed to enrolment. Preliminary data on 148 patients with del 5q31, reported at ASCO 2005 (List *et al*, 2005a, 2005b) included transfusion independence in 64% of patients with a median Hb increase of 3.9 g dl^−1^ and 4-week time to response. Cytogentic response occurred in 76% of patients, with complete cytogenetic remission in 55%. Pathologic complete response in 29%. After a median follow up of 9.3 months, median response duration had not been reached and only 9% of patients had failed therapy. Neutropenia and thrombocytopenia were the most common AEs necessitating treatment interruption/dose reduction.
Lenalidomide	MDS (*n*=215)	Multicentre, USA	II	Ongoing	Closed to enrolment. Data from the 2005 International Symposium on MDS reported transfusion-independence in 21% of 215 patients (ITT) with a median Hb increase of 3.0 g/ dl^−1^. Of 169 low-int-1 risk patients, 25% became transfusion-indepdendent and 43% achieved at least a minor response. Neutropenia (19%) and thrombocytopenia (15%) were the most common adverse effects.
Lenalidomide	MDS (*n*=45)	USA	II	Ongoing	Closed to enrolment. Data presented at the 2005 International Symposium on MDS (Raza *et al*, abstract # P-121) from this study indicated substantial activity in low-risk MDS, including an overall haematological response rate in 43 patients of 56%, with 20 out of 32 transfusion-dependent patients achieving transfusion-independence. Response rate was highest in patients with deletion of 5q.31.1 (83%). Eleven out of 20 patients had cytogenetic remission, including complete cytogenetic remission in 10. Neutropenia and thrombocytopenia were the most common adverse events.
					
*Chronic lymphocytic leukaemia*					
Lenalidomide+rituximab	Chronic lymphocytic leukaemia, relapsed/refractory (*n*=29)	USA	II	Ongoing	Preliminary data from EHA 2005 (Haematologica/The Hematology Journal 2005; 90(Suppl 2): 160, Abstract # 97) on eight patients who completed 30 days of therapy: all eight patients responded with decreases either in ALC or lymph node size. Two patients achieved CR/CRu, two PR and four SD. Since no patients had PD, rituximab was not added. AEs included: grade 3/4 thrombocytopenia (six patients); grade 3/4 neutropenia (three patients); flare reaction (three patients) and tumour lysis syndrome (two patients). Antitumour activity was noted as early as 7 days after therapy.
					
*Other – listed alphabetically by disease, phase*					
Lenalidomide	Glioma, recurrent high-grade (*n*=36)	USA, National Cancer Institute	I	Completed	Preliminary results on the first 18 patients, presented at the 2003 American Society of Clinical Oncology meeting (Fine *et al*, abstract # 418), report that lenalidomide has been well tolerated with only one drug-related toxicity >grade 1 (grade 2 myelosuppression in patient with previous BMT). One patient with rapidly progressive spinal hemangioblastomas, and two patients with rapidly progressive glioblastoma experienced disease stabilisation for 6, 5 and 7 months, respectively.
Lenalidomide	Metastatic malignant melanoma and other advanced solid tumours (*n*= 20)	UK	I	Completed	First published report in solid tumours (Bartlett *et al*, 2004a, 2004b). A total of 17 evaluable patients. One partial response and two clear objective responses, such as resolution of subcutaneous and cutaneous lesions. Evidence of T-cell activation and increased serum IL-12, GM-CSF and TNF-*α* was found. No serious adverse effects were observed.
Lenalidomide	Metastatic malignant melanoma (*n*=295)	Multicentre, (USA, Canada)	III	Completed	Unblinded interim results determined that the trial would not demonstrate a statistically significant treatment effect, although the safety profile was acceptable. On the recommendation of the Data Monitoring Committee, the trial was halted.
Lenalidomide *vs* placebo	Metastatic malignant melanoma (*n*=305)	Multicentre, International	III	Completed	Unblinded interim results determined that the trial would not demonstrate a statistically significant treatment effect, although the safety profile was acceptable. On the recommendation of the Data Monitoring Committee, the trial was halted.
Lenalidomide	Metastatic cancer, refractory (*n*=51)	USA, National Cancer Institute	I	Ongoing	Preliminary results from 2003 meeting of ASCO (Liu *et al*., abstract # 927) on 12 patients (all with metastatic hormone-refractory prostate cancer) report MTD as not yet determined, and administration schedule to be amended. Six out of 12 patients had stable PSA for ⩾8 weeks, no PR or CR. Dose-limiting toxicity at 20 mg day^−1^ was grade 3 thrombosis and grade 3 hypotension.
Lenalidomide	Myelofibrosis with myeloid metaplasia (*n*=27)	USA	II	Ongoing	Preliminary data on the first 15 patients from the 2004 ASH (Tefferi *et al*, 2004) meeting reported substantial biological activity of lenalidomide in MMM, with substantial benefit for a subset of patients. Clinically relevant responses were documented in four out of 15 (27%) patients, including improvement in anaemia (two patients), splenomegaly (two patient), +/or constitutional symptoms (three patients). One patient became transfusion independent; another had Hb increase of >5 g dl^−1^+leucocyte count reduction of >48 × 10^9^ l^−1^. Two patients discontinued due to drug toxicity.
Lenalidomide	Renal cell cancer (*n*=40)	USA	II	Completed	Preliminary data presented at ASCO 2004 (Rawat *et al*, abstract # 4761) reported that lenalidomide was well tolerated with 7.5% PR rate in 36 evaluable patients with progressive, metastatic RCC.
